# Intelligent Medical Garments with Graphene-Functionalized Smart-Cloth ECG Sensors

**DOI:** 10.3390/s17040875

**Published:** 2017-04-16

**Authors:** Murat Kaya Yapici, Tamador Elboshra Alkhidir

**Affiliations:** 1 Faculty of Engineering and Natural Sciences, Sabancı University, Istanbul 34956, Turkey; 2 Sabancı University Nanotechnology Research and Application Center, Istanbul 34956, Turkey; 3 Department of Electrical Engineering, University of Washington, Seattle, WA 98195, USA; 4 Department of Electrical and Computer Engineering, Khalifa University, Abu Dhabi 127788, UAE; tamador.alboshra@gmail.com

**Keywords:** graphene, electrocardiogram, conductive textile, mHealth, ECG electrode, wearable sensor, smart fabric, medical garment, wristband, arrhytmia

## Abstract

Biopotential signals are recorded mostly by using sticky, pre-gelled electrodes, which are not ideal for wearable, point-of-care monitoring where the usability of the personalized medical device depends critically on the level of comfort and wearability of the electrodes. We report a fully-wearable medical garment for mobile monitoring of cardiac biopotentials from the wrists or the neck with minimum restriction to regular clothing habits. The wearable prototype is based on elastic bands with graphene functionalized, textile electrodes and battery-powered, low-cost electronics for signal acquisition and wireless transmission. Comparison of the electrocardiogram (ECG) recordings obtained from the wearable prototype against conventional wet electrodes indicate excellent conformity and spectral coherence among the two signals.

## 1. Introduction

Electrocardiography is a non-invasive method to monitor cardiac activity and can reveal vital information on cardiovascular disorders, including heart rhythm abnormalities, collectively known as arrhythmias or dysrhythmias. Some arrhythmias, such as ventricular fibrillation, are emergency conditions which may lead to sudden cardiac arrest and death. On the other hand, others like atrial fibrillation, although still being serious conditions, are more subtle to detect and could develop over time with prolonged consequences, like blood clotting and stroke [1]. Therefore, to properly interpret the heart activity and diagnose possible rhythm disorders in advance, it is useful to have continuous ECG recordings for extended periods of time. For instance, Holter monitors have been used to record ECG for up to 48 h [2].

Typically, the ECG signal is acquired by mounting conductive biopotential electrodes in multiple locations across the body, and for this purpose Ag/AgCl electrodes have been widely adopted. The conventional Ag/AgCl electrodes, also referred to as “wet” electrodes, contain gel to reduce the skin-electrode contact impedance and are sustained by an adhesive layer to improve the contact to skin. However, in some cases there have been reports of skin irritation and allergic reaction due to long-term use of gel-based “wet” electrodes. In addition, the electrode performance degrades with time due to drying of the gel [3]. Therefore, standard Ag/AgCl electrodes are not favorable for long-term monitoring applications. Instead, “dry” electrodes freed from gel have been sought after, which are more suitable for long-term monitoring applications and can provide the desired comfort level to be continuously worn as part of wearable health monitoring devices.

So far, different types of dry electrodes have been reported which can be categorized into two types as contact and non-contact electrodes. In contact electrodes, direct physical coupling is established between the skin and the electrode, where the coupling efficiency may be further enhanced by the presence of sweat or moisture [4,5]. On the other hand, non-contact operation relies on capacitive coupling between the electrode and the skin via separation by dielectric material or air [6]. Examples of dry electrodes include microneedle structures based on silicon micromachining [7] and 3D printing technology [8], flexible sensors with thin-film metallic coatings [9], nanomaterial-doped polymer electrodes [10], and capacitive electrodes built on printed circuit boards (PCB) [11].

An alternative technology to develop dry electrodes is based on utilizing conductive textiles. Textiles are one of the most frequently used materials in daily life, and they are ideal for building wearable health monitoring devices due to their inherent flexibility, and soft and comfortable texture. Moreover, electronic components can be readily attached to conductive textiles, which greatly simplifies the development of wearable, intelligent medical garments for long-term monitoring of essential physiological signals. The seamless integration of textile-based biopotential electrodes to low-power, small-form-factor wireless transmission modules and handheld devices would make remote cardiac monitoring possible with minimal disturbance to the daily routine of the patient. To this end, the abundance and variety of clothing accessories such as snap fasteners, elastic belts, and bands further support the integration of electronics on textiles to realize wearable medical garments. For instance, tight vests, belts and shirts worn around the upper chest region were used to sense ECG signals with the help of the textile-based electrodes embedded inside the garment [12,13,14,15,16,17].

Some ideal properties of conductive textiles for wearable garments include high electrical conductivity, high mechanical durability and stability against repeated use, a high degree of comfort and flexibility, low-cost, and large-area manufacturability. To realize conductive textile electrodes, different fabrication methods have been investigated, including screen printing techniques [18,19], metallic threading [20,21], the addition of conductive polymers to fibers [22], metal coating by physical vapor deposition [23], and electroplating [24]. In an effort to further improve conductive textile technology and its applications, we have recently merged the outstanding material properties of graphene with soft fabrics using a low-cost, scalable technology and demonstrated graphene-clad textile biopotential electrodes with excellent performance [25].

In this paper, we capitalize upon the superior properties of graphene-clad conductive textiles and report the development of a fully-wearable, cloth-based, smart medical garment for ECG monitoring. The prototype is based on elastic bands which house built-in graphene-clad textiles as the ECG sensing electrodes, electronic circuitry for ECG readout and transmission, and lithium-ion batteries for power. We demonstrate successful ECG signal acquisition, processing, and wireless transmission, all achieved on the same wearable textile platform. The unique system-level design of the prototype allows ECG monitoring using minimal number of elegant clothing accessories to be comfortably worn around the wrists or neck, and alleviate the shortcomings of conventional wet-based electrodes in long-term monitoring applications.

## 2. Materials and Methods

### 2.1. Preparation and Integration of Graphene-Clad Textiles into Wearable Garments

**Figure 1:**
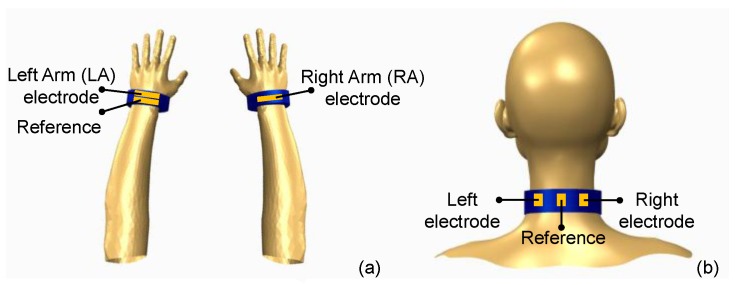
System level integration and placement of textile-based electrodes to form wearable, intelligent, medical garments for ECG sensing from the wrist or the neck: (**a**) wristband; (**b**) neckband.

In typical twelve-lead electrocardiography ten conductive electrodes are placed across the chest and limbs; however, for ambulatory monitoring or wearable applications, as few as three electrodes may be used to acquire the ECG signal [26]. In this work, two types of textile-based, wearable ECG sensors were developed as demonstrators of intelligent medical clothing in the form of a wristband and neckband which can be easily carried on the body. In the case of wristbands, ECG measurement is achieved by placing two wristbands, one on each wrist (Figure 1a). Each wristband contains a single sensing electrode, with one of the wristbands containing an additional electrode as a reference node. On the other hand, a neckband, as illustrated in (Figure 1b), is constructed by adding three pieces of conductive textile, out of which two serve as sensing electrodes and one as a reference electrode.

Textile-based, dry electrodes were prepared by dipping an ordinary piece of nylon textile into a graphene oxide (GO) suspension, which was arranged ahead of time by following the protocol for the modified Hummers’ method. Then, the dipped textile was thermally treated to allow conformal cladding of GO flakes on individual fibers of the textile. The GO-clad textile was then dipped into hydrogen iodide (HI) to reduce and convert the graphene oxide to graphene, which ultimately results in graphene-clad textiles with higher conductivity. Nylon was chosen as the textile material due to its low surface roughness, which in turn promotes higher conductivity upon coating with graphene. Detailed description of the preparation of graphene-clad textiles was reported earlier in [25].

**Figure 2:**
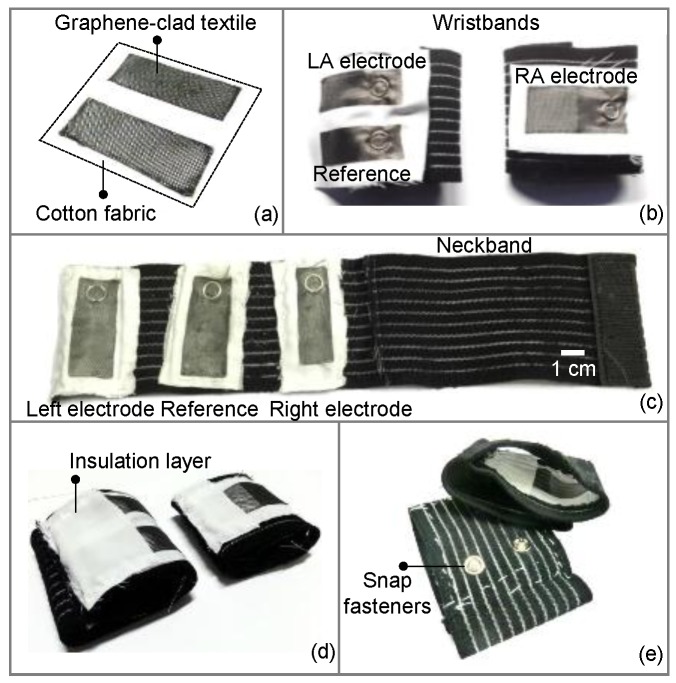
Steps showing the integration of graphene-clad textiles into elastic bands: (**a**) textile pieces glued on a cotton sheet; (**b**) sewing the textiles to form wristband; and (**c**) neckband; (**d**) insulation of snap fasteners (**e**) prototype of a complete graphene-clad textile integrated wristband.

The prepared samples of graphene-clad textile were cut into smaller sizes of approximately 6 cm × 3 cm to form the ECG electrodes. The electrodes were subsequently glued to a larger piece of cotton fabric (Figure 2a), which allows easy attachment of electrodes to various types of clothing by sewing with thread. With this approach, electrodes function as disposable parts of the wearable garment and can be replaced as needed to maintain system performance. To construct the wearable medical garment, elastic bands were chosen; as they ensured firm coupling to the skin for high fidelity ECG acquisition and helped minimize the susceptibility to possible signal distortions in case the patient is in physical motion. Figure 2b shows a pair of wristbands, with the wristband for the left arm having two electrodes (one of them for the reference node) separated by 1 cm, and the wristband for the right arm has one electrode. Similarly, three textile electrodes separated apart by 3 cm were attached to a neckband, where the middle electrode was used as a reference node (Figure 2c). The electrodes and elastic bands were sandwiched between metallic snap fasteners to establish an electrical link between the interior of the band facing the skin, to the exterior for interfacing with circuitry. Finally, the faces of the snap fasteners on the inner sides of the bands were covered and insulated with cotton fabric, in order to avoid direct contact of metal pieces to skin (Figure 2d,e). This effectively created an electrode contact area of ~6 cm^2^ comparable to standard electrode dimensions.

### 2.2. Performance Evaluation

**Figure 3:**
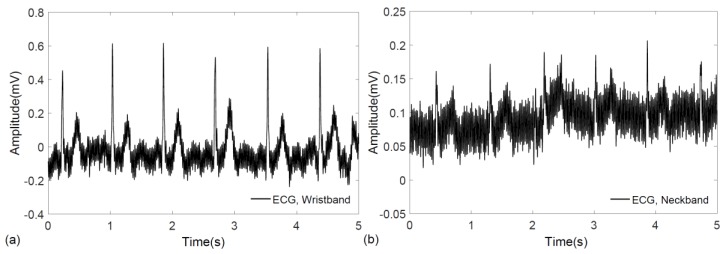
Raw electrocardiograms recorded using a commercial data acquisition unit. (**a**) ECG signal obtained from the wearable wristband; and (**b**) ECG signal obtained from the wearable neckband.

To evaluate the performance of the wearable elastic bands in biopotential signal acquisition, electrocardiogram measurements were performed on a voluntary, healthy subject using a commercial data acquisition system (PowerLab 26T, AD Instruments, Dunedin, New Zealand). The subjects gave their informed consent for inclusion before they participated in the study, and the experimental procedures involving human subjects described in this paper followed the principles outlined in the Declaration of Helsinki. Signals from the wrist and neck were recorded successfully with no prior skin preparation or application of conductive gels. In case of ECG measurement from the wrist, lead-I configuration was followed, where two bands, one on each hand, were fastened by Velcro. Similarly, the ECG signal was also recorded by wrapping a single elastic band around the neck, where the positions of the sensing electrodes fell roughly below the ear, around the carotid arteries. The raw data for ECG signals obtained from the wrist and neck are shown in Figure 3a,b, respectively.

A typical electrocardiogram is likely to be distorted with different noise and interference sources such as power-line noise (50/60 Hz) and motion artifacts. Therefore, noise filtering techniques are needed to obtain a clean ECG signal for proper medical interpretation. For filtering, we have applied the discrete wavelet transform (DWT) on the raw ECG recordings. Discrete wavelet transform (DWT) serves as a beneficial filtering tool for ECG, as it allows the signal to be decomposed into different frequency bands and, therefore, permits easy identification of noise patterns which usually fall within characteristic frequency bands [27]. In doing so, we have decomposed the sampled signals using a tree-structure filter bank with 10 levels using Daubechies wavelet families, as they are frequently used in ECG processing [27]. At each level, the incoming signal was passed through a low-pass and a high-pass filter and outputs from each were down-sampled by a factor of two. Following this, the unwanted frequency bands were eliminated from the ECG signal which resulted in denoising and improvement of the signal-to-noise ratio.

**Figure 4:**
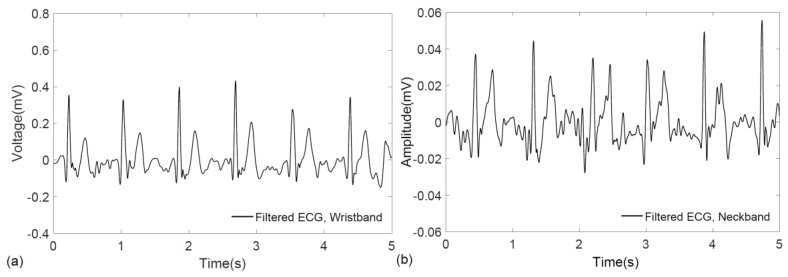
Filtered ECG recordings obtained from (**a**) wristband; and (**b**) neckband.

The implementation of discrete wavelet transform to denoise the electrocardiogram was performed in MATLAB^®^ (Mathworks, Natick, MA, USA). Figure 4a,b show the filtered ECG signals from the wristband and neckband, respectively. After filtering, the QRS complex, which is a distinctive feature of an ECG signal, is revealed in both of the signals where the wristband displays a stronger signal than the neckband. The difference in ECG amplitude is attributed to the larger biopotential difference occurring between reciprocal electrodes placed on the two wrists compared to those placed around the neck with small separation.

### 2.3. System-Level Design

**Figure 5:**
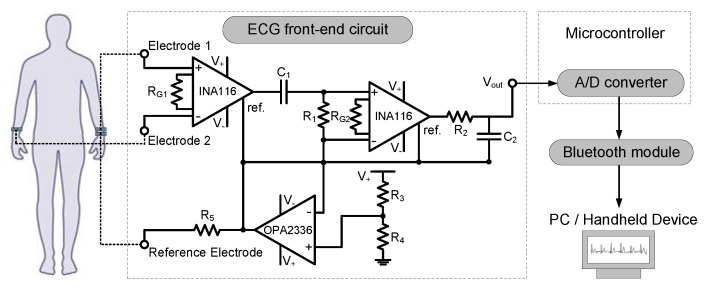
Block diagram of the graphene-clad textile based wearable ECG monitoring system and schematic of the ECG front-end acquisition circuit.

Upon verifying the performance of ECG measurement using commercial data acquisition unit, a complete, wearable prototype of a graphene-clad textile embedded band with integrated electronics was developed. For this purpose, low-cost, commercially available discrete components were used, and the ECG acquisition circuitry was custom-designed. The system-level block diagram of the wearable prototype is shown in Figure 5, which is comprised of elastic bands embedded with graphene-clad textiles, front-end ECG acquisition circuitry, microcontroller, and Bluetooth module. Ideally, ECG acquisition circuitry intended for wearable health monitoring applications should deliver high performance with a small footprint and with minimum power consumption. Therefore, all sub-blocks were designed or selected with the goal to minimize the total size to fit on elastic bands, and wristbands were selected as a demonstrator.

To pick-up the small biopotentials occurring during the course of normal cardiac activity, a front-end ECG acquisition circuit, essentially a differential amplifier with high gain, is needed. The schematic of the ECG front-end circuit shown in Figure 5 consists of different blocks responsible for amplification, analog filtering and suppression of common-mode signals. After amplifying and filtering the ECG signal, an analog to digital (A/D) convertor was used to digitize the acquired signal to be sent via a Bluetooth module to a remote PC where offline signal processing and characterization can be performed. This post-processing approach further eliminates the need for excessive electronic components that would be required for comprehensive on-board filtering, which subsequently result in total reduction of the circuit size and power consumption.

Since ECG signals are relatively weak and contaminated by various noise sources, proper signal amplification with minimum noise is needed for successful acquisition of biopotentials. To this end, instrumentation amplifiers are widely employed and constitute an important part of the ECG front-end circuit. For robust and high performance circuit operation, the instrumentation amplifier should be carefully selected based on several specifications. First, the instrumentation amplifier should have high input impedance to minimize the input current offset and therefore reduce the voltage noise at the output [28,29]. Additionally, a small input current noise is desirable for low-noise amplification, and a high common-mode rejection ratio (CMRR) is helpful to mitigate common mode noises, such as power-line noise and electromagnetic interference [30]. Considering these factors, we chose an instrumentation amplifier (INA116, Texas Instruments, Dallas, TX, USA) with a high CMRR of ~84 dB, a low input current noise density of 0.1 fA/√Hz and high input impedance of 10^15^/0.2 Ω/pF [29].

Another important consideration in the design of ECG front-end circuit is regarding the filtering of primary noise components. ECG signals are severely affected by the interference at power-line frequencies of 50 or 60 Hz. Therefore, we have designed a cascaded bandpass filter, which allows frequencies in the 0.2 to 40 Hz range relevant to ECG signals to pass, while blocking power-line and high-frequency noise components. To achieve the above passband behavior, the value of R_1_ and C_1_ were configured to 100 kΩ and 6.8 µF creating a high-pass filter, whereas R_2_ and C_2_ value were set to 39 kΩ and 0.1 µF to implement a low-pass filter.

Additionally, the amplification of the ECG signal was divided between two stages to prevent output saturation. Considering the range of suitable input voltages for the A/D converter, the amplifier gain ($G=1+\frac{50\;kΩ}{R_{G}}$) [29] for each stage was set to G = 51 by selecting the values of R_G1_ and R_G2_ as 1 kΩ; which resulted in a total gain of ~68 dB. Alongside the gain and filtering blocks, another common circuit configuration known as the driven-right leg (DRL) was implemented to achieve single supply, battery-powered circuit operation and to further enhance the common-mode rejection [31]. The DRL circuit was formed by a voltage divider where the resistors R_3_ and R_4_ were set equal to provide a virtual ground voltage at V_+_/2. This voltage was coupled to a buffer amplifier which was constructed using a standard op-amp chip (OPA2336, Texas Instruments, Dallas, TX, USA), and the output of the buffer was fed to the human body through a limiting resistor (R_5_) and reference electrode to improve the common-mode rejection.

The acquired ECG signal was sampled at 500 Hz by the built-in, 10-bit analog-to-digital converter of a commercial microcontroller board (Arduino Pro Mini ATmega328, Arduino, Italy), and transmitted via a Bluetooth module (BlueSMiRF Silver, SparkFun, Boulder, CO, USA) to a personal computer. The data rate for Bluetooth transmission was set to 57.6 kbps to ensure a total sampling rate of 500 Hz. The Bluetooth module was interfaced with the computer through RS-232 communication and then the received ECG signal was further filtered by MATLAB^®^. A lithium-ion polymer battery with 3.7 volts and 2000 mAh capacity was used to power both the ECG front-end circuitry and the Bluetooth module. The specifications of all system blocks and component values are summarized in Table 1.

## 3. Results

**Figure 6:**
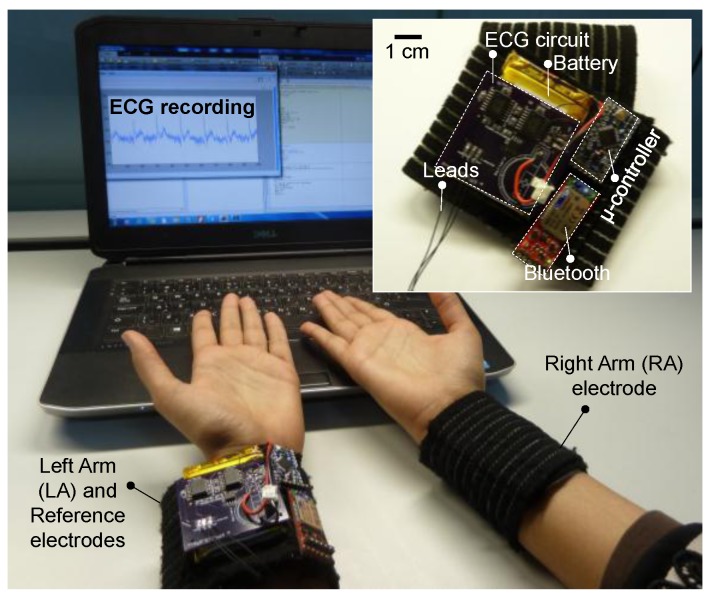
Live demonstration of ECG measurement using the prototype graphene-clad textile embedded wearable wristband with integrated electronics.

Using surface mount electronic components, the entire front-end circuit was built on a standard two-layer PCB with approximate dimensions of 5 cm × 5 cm, and thin wires were used to electrically interface the circuit with the microcontroller and Bluetooth module. All system parts were neatly fixed onto one of the wristbands by directly sewing with thread through access holes drilled on the circuit boards. Figure 6 shows the prototype of the wearable, graphene-clad textile embedded wristbands with integrated electronics for ECG monitoring.

**Figure 7:**
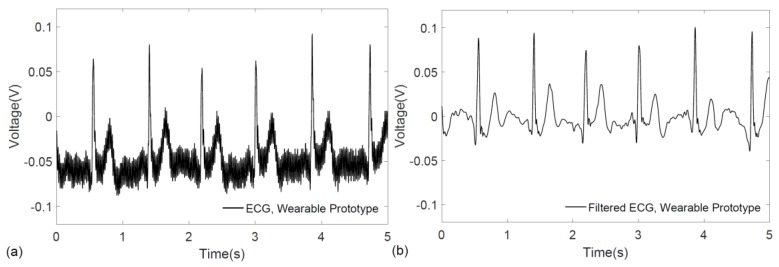
ECG signal acquired from the wrists using the prototype graphene-clad textile embedded wearable garment: (**a**) raw ECG recording; and (**b**) filtered ECG signal.

The functionality of the prototype system was verified on a voluntary subject by real-time monitoring and transmission of their ECG signals to a personal computer, where the raw ECG recording is shown in Figure 7a. For accurate visualization of ECG patterns, the signal was further denoised by applying the discrete wavelet transform in MATLAB^®^. As illustrated in Figure 7b, the characteristic QRS complex is easily identified in the filtered ECG signal.

**Figure 8:**
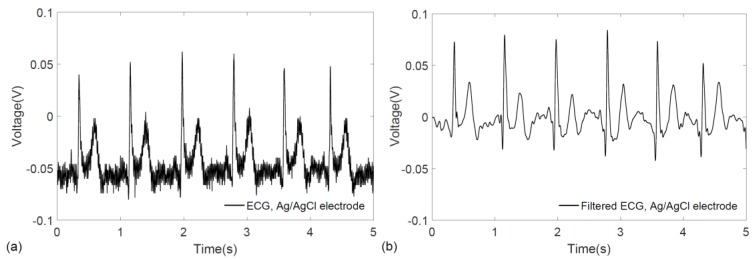
ECG signal acquired from the wrists using conventional Ag/AgCl electrodes with the same: (**a**) raw ECG recording; and (**b**) filtered ECG signal.

To further benchmark the performance of the wearable prototype and compare it with conventional pre-gelled electrodes, ECG signals were acquired using Ag/AgCl (3M^TM^ Red Dot^TM^ 2560, 3M, St. Paul, MN, USA) electrodes while keeping the same experimental conditions including the measurement locations (left and right wrist) and the measurement circuitry. Since the miniaturization of the overall system is a key design consideration for wearability, the front-end circuit was developed for single channel data acquisition rather than simultaneous recording from multiple sources. Therefore, to record the ECG biopotentials from the same location and with the same circuit, the electrocardiograms were acquired immediately after one another with a gap of less than two minutes, which also ensured that no significant physiological changes occurred in the healthy test subject. Figure 8a,b show the raw and filtered ECG signals obtained with conventional electrodes. For the filtering of signals obtained from conventional Ag/AgCl electrodes, the same DWT procedure outlined earlier was followed.

**Figure 9:**
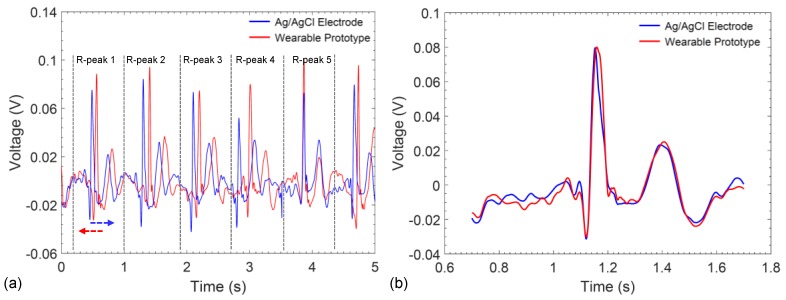
(**a**) ECG segmentation and alignment of P-QRS-T intervals recorded from conventional Ag/AgCl electrodes and the graphene-clad textile embedded wearable prototype; (**b**) example of an aligned P-QRS-T interval.

Even though the gap between subsequent ECG recordings is small, the existence of a time-delay prevents direct comparison of the signals in the time-domain. Therefore, to gain an understanding of the correlation between the recorded signals, ECG waveforms recorded from the Ag/AgCl electrodes and the wearable prototype were divided into P-QRS-T intervals (Figure 9a) and shifted in the time-axis to align the R-peaks (Figure 9b). Then, the cross-correlation between all 25 possible combinations of P-QRS-T segments were calculated. Comparison of the results of conventional electrodes with the wearable wristband show that the signals conform very well in time domain and display an average cross-correlation of 88% for the entire waveform and a maximum of 97% was achieved between two P-QRS-T segments (Table 2).

**Figure 10:**
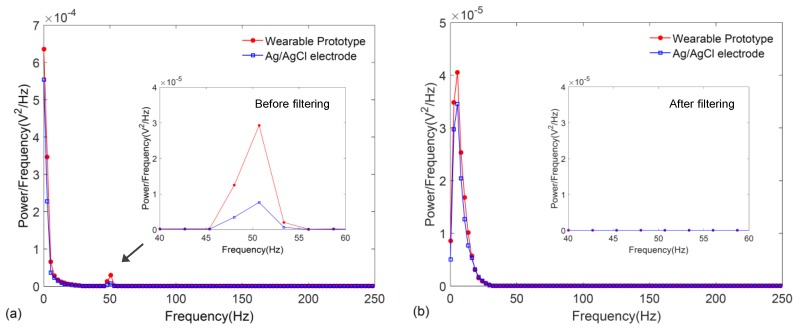
Power spectral density of the ECG signals obtained from the graphene-clad textile embedded wearable prototype and conventional Ag/AgCl electrodes: (**a**) before filtering; and (**b**) after filtering.

On the other hand, frequency response characteristics of the signals were also compared by plotting their power spectra which were estimated by using the Welch periodogram with a Hamming window available in MATLAB^®^. As illustrated in Figure 10a,b, the power spectrum of the ECG signal obtained from the wearable wristband is in excellent agreement to that of the Ag/AgCl electrodes both before and after filtering, where the critical QRS morphology of the ECG lying in the 0–30 Hz frequency range is accurately captured. As shown in the insets, the filtering effectively removes the 50 Hz interference.

## 4. Discussion

Another important parameter for wearable ECG monitoring applications is robustness of the electrode and signal acquisition system against physical motion. Textile-based ECG electrodes are known to be prone to motion artifacts, and for this reason we have previously reported a simple adaptive filtering approach for the removal of motion artifacts [32]. The reason for such artifacts have been primarily attributed to the displacements between the textile electrode and the skin which effectively cause variations in the electrode-skin impedance and epidermal biopotentials [33]. In the developed prototype, we have noticed that the susceptibility to motion artifacts is significantly compensated with the use of elastic bands. This is mainly due to the tight-fitting pressure applied on the skin by the wristbands, where the applied pressure helps maintain a stable electrode-skin contact and also lowers the electrode-skin contact impedance by reducing the air gap between the textile electrodes and the skin [33].

**Figure 11:**
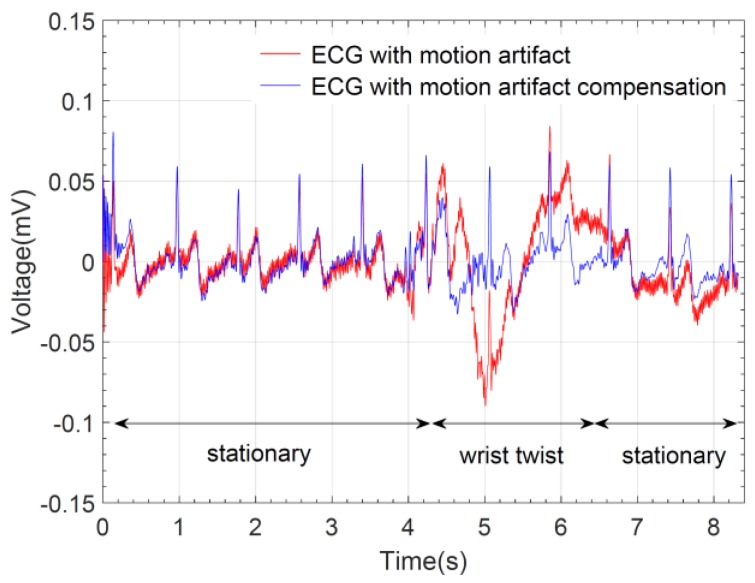
Electrocardiogram recording showing motion-related artifacts as a result of twisting of the wrist and artifact removal by adaptive filtering.

The elastic bands physically limit the displacement of the graphene textile electrodes to a large extent, when they are subject to pure lateral and vertical motions. Therefore, the susceptibility of the wearable prototype to motion artifacts along these directions are minimized. However, in more complex actions such as twisting of the wrist, the resulting torsional strain would directly affect the stability of the electrode-skin interface and motion artifacts may be more pronounced. Hence, to evaluate the response of the graphene textile embedded ECG wristband against motion artifacts, we have performed electrocardiogram recordings when the wrist is twisted in a circular pattern. The red trace in Figure 11 shows an electrocardiogram recorded for a duration of eight seconds in which during the initial four seconds the wrist is in stationary condition and the typical ECG pattern with the P-QRS-T complex is clearly distinguished. This is followed by a rapid twisting of the wrist for a duration of approximately two seconds which caused artifacts and distortions in the signal. After termination of the wrist motion, the electrocardiogram assumes its normal pattern.

To compensate for the motion artifacts, we have applied an adaptive filtering algorithm [32] which required acquisition of reference signals that are highly correlated with the motion. For this purpose, two additional graphene textile electrodes were placed inside wristbands and signals were acquired using a data acquisition unit. The blue trace in Figure 11 shows the electrocardiogram where the distortions that occurred during twisting of the wrist are removed. Effectively, the elastic band-based unique design of our wearable prototype allows for the minimization of possible distortions under the presence of motion and coupled with the wireless data transmission capability further post-processing can be done to achieve high quality ECG acquisition. Real-time removal of distortions due to motion artifacts, baseline wander, power-line or high-frequency noise could also be possible by embedding the related filtering algorithms to the on-board microprocessor in the prototype system.

The graphene textile embedded wristbands provided stable electrocardiogram recordings throughout the measurements performed in this study which lasted for a duration of approximately one month. We anticipate that, with time, the graphene textiles embedded inside the wristbands could wear out, in which case either the graphene textiles or the entire wristband (excluding the electronics) could be readily replaced at a low cost. Owing to the scalable fabrication and integration of our graphene textiles into clothing, the electrode size can also be readily increased to adjust the electrode-skin contact impedance. Our measurements show that the average impedance values of the graphene textile embedded wristband ranges from 87.5 kΩ to 55 kΩ in the 10–50 Hz range, which are in good agreement with our measurements on conventional Ag/AgCl electrodes having impedance values from 50.9 kΩ to 20 kΩ in the same frequency range. With increasing electrode size it could be possible to further reduce the contact impedance [34], which would especially be helpful to reduce the burden on the input stage of the analog front-end.

## 5. Conclusions

In this work, we have developed a complete prototype of a wearable garment with integrated electronics for electrocardiogram monitoring. As ECG electrodes, we have employed graphene-clad conductive textiles which are soft and wearable, washable, weavable, and manufactured using a low-cost, scalable technology. The textile electrodes were stitched inside elastic bands for easy attachment to body locations, such as the wrist and the neck, and ECG signals were successfully acquired with minimal disruption to usual dressing habits. A battery-powered prototype was built on wristbands which housed all of the electronic circuitry needed for portable acquisition and wireless transmission of ECG signals to a remote personal computer. The performance of the prototype wearable ECG wristband is in excellent match to that of conventional Ag/AgCl electrodes; with the added benefit of not requiring prior skin preparation or the application of gels.

We envision the possible application areas of this prototype to include stationary ECG monitoring for in-bed patients at home or possibly at a hospital, as well as monitoring during physical activity, which we are planning to investigate on a larger pool of subjects in future studies. The graphene-clad, textile-based prototype medical garments can be further developed for application to other biopotential monitoring procedures including EEG (electroencephalography) and EMG (electromyography).

## Figures and Tables

**Table 1:** System blocks and specifications.

Parts	Specifications
Bluethooth module	Power consumption: 26 µA sleep, 3 mA connected, 30 mA transmit
(BlueSMiRF Silver)	Baud rate: 57,600
	Bandwidth: 0.2–44 Hz
	Gain: 68 dB
ECG front-end circuit	Power supply: 3.3–5 V
	R_G1_–R_G2_, R_1_, R_2_, R_3_–R_5_: 1 kΩ, 100 kΩ, 39 kΩ, 100 kΩ
	C_1_, C_2_: 6.8 µF, 0.1 µF
A/D converter	Resolution: 10 bit
(Arduino Pro Mini)	Power supply: 3.3–5 V

**Table 2:** Cross-correlation values among all possible P-QRS-T segment pairs.

Ag/AgCl	Wearable Prototype				
	R-peak 1	R-peak 2	R-peak 3	R-peak 4	R-peak 5
R-peak 1	81.9%	94.4%	95.0%	92.3%	84.5%
R-peak 2	83.5%	87.4%	81.0%	97.0%	85.5%
R-peak 3	93.3%	89.9%	82.1%	91.7%	92.9%
R-peak 4	89.3%	92.8%	89.4%	81.7%	91.8%
R-peak 5	89.0%	89.9%	84.3%	81.3%	84.4%
**Average:**	88.3%			**Maximum:**	97.0%
